# Voxel-based dosimetry predicting treatment response and related toxicity in HCC patients treated with resin-based Y90 radioembolization: a prospective, single-arm study

**DOI:** 10.1007/s00259-023-06111-9

**Published:** 2023-01-18

**Authors:** Nima Kokabi, Linzi Arndt-Webster, Bernard Chen, David Brandon, Ila Sethi, Amir Davarpanahfakhr, James Galt, Mohammad Elsayed, Zachary Bercu, Mircea Cristescu, S. Cheenu Kappadath, David M. Schuster

**Affiliations:** 1 Division of Interventional Radiology and Image-Guided Medicine, Department of Radiology and Imaging Sciences, Emory University School of Medicine, Atlanta, GA USA; 2 Division of Interventional Radiology, Department of Radiology, Mount Sinai School of Medicine, New York, NY USA; 3 Division of Interventional Radiology, Department of Radiology, University of Texas at San Antonio, San Antonio, TX USA; 4 Division of Nuclear Medicine and Molecular Imaging, Department of Radiology and Imaging Sciences, Emory University School of Medicine, Atlanta, GA USA; 5 Division of Abdominal Imaging, Department of Radiology and Imaging Sciences, Emory University School of Medicine, Atlanta, GA USA; 6 Division of Interventional Radiology, Department of Radiology, Memorial Sloan Kettering Cancer Center, New York, NY USA; 7 Division of Interventional Radiology, Department of Radiology, Medical College of Wisconsin, Milwaukee, WI USA; 8 Division of Interventional Radiology, Department of Radiology, University of Texas MD Anderson Cancer Center, Houston, TX USA

**Keywords:** HCC, Yttrium-90, Resin microspheres, Tumor dose response threshold, Liver dose toxicity threshold

## Abstract

**Background:**

There is an increasing body of evidence indicating Y90 dose thresholds for tumor response and treatment-related toxicity. These thresholds are poorly studied in resin Y90, particularly in hepatocellular carcinoma (HCC).

**Purpose:**

To evaluate the efficacy of prospective voxel-based dosimetry for predicting treatment response and adverse events (AEs) in patients with HCC undergoing resin-based Y90 radioembolization.

**Materials and methods:**

This correlative study was based on a prospective single-arm clinical trial (NCT04172714), which evaluated the efficacy of low/scout (555 MBq) activity of resin-based Y90 for treatment planning. Partition model was used with goal of tumor dose (TD) > 200 Gy and non-tumoral liver dose (NTLD) < 70 Gy for non-segmental therapies. Single compartment dose of 200 Gy was used for segmentectomies. Prescribed Y90 activity minus scout activity was administered for therapeutic Y90 followed by Y90-PET/CT. Sureplan® (MIM Software, Cleveland, OH) was used for dosimetry analysis. Treatment response was evaluated at 3 and 6 months. Receiver operating characteristic curve determined TD response threshold for objective response (OR) and complete response (CR) as well as non-tumor liver dose (NTLD) threshold that predicted AEs.

**Results:**

*N* = 30 patients were treated with 33 tumors (19 segmental and 14 non-segmental). One patient died before the first imaging, and clinical follow-up was excluded from this analysis. Overall, 26 (81%) of the tumors had an OR and 23 (72%) had a CR. A mean TD of 253 Gy predicted an OR with 92% sensitivity and 83% specificity (area under the curve (AUC = 0.929, *p* < 0.001). A mean TD of 337 Gy predicted a CR with 83% sensitivity and 89% specificity (AUC = 0.845, *p* < 0.001). A mean NTLD of 81 and 87 Gy predicted grade 3 AEs with 100% sensitivity and 100% specificity in the non-segmental cohort at 3- and 6-month post Y90, respectively.

**Conclusion:**

In patients with HCC undergoing resin-based Y90, there are dose response and dose toxicity thresholds directly affecting outcomes.

Clinical trial number: NCT04172714.

## Introduction

Yttrium 90 (Y90) radioembolization is a locoregional therapy that targets liver malignancies through the use of microspheres injected into the tumoral arterial bed [1]. Crucial to Y90 therapy is ensuring the tumor receives sufficient radiation to induce pathological necrosis. Patient survival has been shown to improve with personalized dosimetry that maximizes tumor dose [2–4]. With recent studies showing tumor dose thresholds of > 205 Gy [2] and > 400 Gy[5] in patients with hepatocellular carcinoma (HCC) treated with glass-based microspheres resulting in improving survival and tumor response, the field of dosimetry for Y90 radioembolization to treat liver malignancies is rapidly evolving. Nevertheless, the tumor dose response threshold for HCC treated with resin-based Y90 remains a gap in knowledge.

Additionally, with the escalation of tumor dose, radiation delivery to the rest of the liver and lungs may cause greater adverse events (AEs) [6]. Mild and often transient clinical and laboratory toxicities are common after Y90 radioembolization, most commonly fatigue and abdominal pain [7], as well as transaminitis and hyperbilirubinemia [8]. Treatment-related AEs are more frequent, with lobar/non-segmental therapies with radiation segmentectomy thought to have less frequent AEs. However, a recent prospective trial (RASER study) evaluating the efficacy of glass Y90 radiation segmentectomy in HCC reported 28% grade 3 treatment-related AEs [3]. Despite frequent, albeit mostly mild AEs, there is a paucity of prospective data that describes non-tumoral liver dose (NTLD) thresholds at which AEs occur for both glass and resin Y90. A recent retrospective, multicenter investigation was unable to find a non-tumoral liver dose threshold that could predict hyperbilirubinemia in 209 patients with unresectable HCC who were treated with glass Y90 [4].

The aim of this prospective trial was twofold: (1) to determine a tumor dose (TD) threshold that predicts complete and objective tumor response and (2) to determine a NTLD threshold that can predict severe treatment-related toxicities at 3 and 6 months after resin-based Y90 radioembolization in patients with HCC.

## Materials and methods

### Study design

In a prospective single-arm clinical trial (NCT04172714), which evaluated the efficacy of low/scout activity of resin-based Y90 for treatment planning, the secondary aim of the study was to determine dose thresholds for treatment response and toxicity. The study was approved by the local institutional review board and is Health Insurance Portability and Accountability Act compliant. From December 2019 to January 2021, eligible participants with treatment-naïve HCC were recruited after obtaining informed written consent. All participants recruited were deemed to potentially benefit from Y90 radioembolization for downstaging therapy or as definitive therapy by the local institutional multidisciplinary tumor board.

Inclusion criteria were adults deemed to benefit from Y90, presence of HCC confirmed by Liver Reporting and Data System (LIRADS) on CT or MRI or biopsy, less than or equal to 3 lesions confined to a single lobe, targeted tumors measuring ≥ 2 cm and ≤ 8 cm, absence of extrahepatic metastasis, Eastern Cooperative Oncology Group (ECOG) status ≤ 2, and life expectancy ≥ 6 months.Required laboratory values were bilirubin < 2 mg/dL for non-segmental therapy or < 3 mg/dL for segmental therapy, albumin > 3 g/dL, international normalized ratio (INR) < 2, AST/ALT three times the upper limits of normal, and platelet count > 50,000/mcL.

Exclusion criteria were patients who were found to have extrahepatic disease during mapping, those who had a lung shunt fraction that would result in dose modification and cause inadequate tumoral dose, and those with a history of systemic or liver-directed therapy for HCC.

### Angiographic mapping procedure

All procedures were performed by interventional radiologists with authorized user status and over 5 years of experience with radioembolization (NK, ZB). All patients underwent angiographic mapping with visceral angiography to assess hepatic arterial tumor supply and evaluated for any extrahepatic shunting [9]. The trial steps were explained in detail in a recent publication by the authors, which illustrated the efficacy and safety of scout resin Y90 for radioembolization treatment planning [10]. Briefly, the procedure was performed by first obtaining radial or femoral access. Then, a 5-French catheter and a co-axial microcatheter were used to selectively identify the vessel(s) supplying the tumor(s). From that location, cone beam computed tomography (CBCT) with 3D reconstruction was used to evaluate blood supply to the targeted tumor(s) and ensure complete tumor perfusion. Coil or plug embolization was performed to occlude branch vessels deemed by the operator to be high risk for non-target embolization. From the selective microcatheter location that perfused the entirety of the tumor, 148 MBq of Tc99m MAA was administered for non-segmental therapy (2 adjacent segments or a liver lobe), and 74 MBq was administered for segmental therapy. Catheters were removed, and the vascular sheath at the access site was secured to allow the participant to be transferred to nuclear medicine to undergo Tc99m MAA planar and single-photon emission computed tomography (SPECT)/CT scans (Discovery 670, GE, Haifa, Israel).

The patients were then brought back to interventional radiology for the second mapping procedure. Using the same techniques as above, a second mapping was performed using resin-based Y90 microspheres (Sirtex Medical, Woburn, MA, USA). Using the same catheters and microcatheters, and from the same microcatheter location from which MAA was administered, 555 MBq of 3-day precalibrated Y90 resin microspheres were administered as the scout dose (ScoutY90). If there was dual blood supply to the tumor, 10 mCi 37 MBq of Y90 was administered to each artery. This activity of 15 mCi was determined based on the author’s prior work demonstrating 481 MBq was sufficient for accurate imaging on both SPECT and position emission tomography (PET) [11].

After the second mapping procedure, all catheters and sheaths were removed, and hemostasis was achieved with a trans-radial band or a femoral artery closure device. The patient was then transferred to nuclear medicine for Y90 PET/CT (Vision 600, Siemens, Hoffmann Estates, IL, USA). After discharge criteria were met, all patients were discharged home on the same day as their procedure.

### Dosimetry analysis

Nuclear medicine–trained physicians with over 10 years of experience performed all imaging evaluations (DB, IS, and DS). Image acquisition and quality control were performed under the supervision of a PhD-trained medical physicist with greater than 30 years of experience (JG). After MAA delivery, all the patients underwent lung shunt fraction (LSF) evaluation using planar and SPECT/CT [12–16]. Semi-automated segmentation was performed with MIM v6.9 (MIM Software, Columbus, OH, USA) [17]. Tc99 MAA tumor to normal ratio (TNR) was also calculated by using the respective volume and activity counts of the tumor and non-tumoral contoured liver. LSF and TNR for the scout dose were assessed with PET/CT [18] with the same method described above. Post Y90 dosimetry analysis was performed using voxel-based dosimetry by the MIM Sureplan® software. The dosimetric variables measures were mean TD to the entire tumor volume, minimum TD, and mean tumor dose to top 30% (TD-V30) and 70% (TD-V70) of tumor volume. NTLD was calculated by averaging the absorbed dose over the treated non-tumor volume.

### Prospective dosimetry treatment planning

Therapeutic Y90 activity was calculated based on Tc99 MAA TNR and LSF from SPECT/CT as the current standard of care. Utilizing the partition model, patients undergoing non-segmental (lobar or 2 adjacent segments) Y90 therapies had their treatment planning with the goal to deliver > 200 Gy to the tumor and < 70 Gy to the non-tumoral liver [19]. For those undergoing segmental therapies, a single compartment target dose of 200 Gy was used. The decision on segmental and non-segmental therapies was made by the treating interventional radiologists based on whether the targeted tumor angiosome was confined to 1 hepatic segment (segmentectomy candidate) versus greater than 1 hepatic segment. Prescribed Y90 activity minus the scout dose activity was administered 3 days post mapping. All dosimetry planning was calculated using MIM v6.9 (MIM Software, Cleveland, OH, USA) by experienced interventional radiologists and nuclear medicine physicians [17]. No patients were excluded from this study due to lack of ability to deliver > 200 Gy to the tumor and < 70 Gy to NTLD while planning.

### Therapeutic radioembolization procedure

Three days after mapping, participants returned to the interventional radiology suite for outpatient therapeutic Y90 administration. The procedure was identical to the mapping procedure, but participants were treated with therapeutic dose Y90 administered. Three-day pre-calibrated resin Y90 microspheres were used for segmentectomies, and 2-day pre-calibrated Y90 microspheres were used for non-segmental therapy. The patients then undergo Y90 PET/CT [18, 20–23]. All the patients were discharged on the same day.

### Clinical outcome measurement

Clinical and biochemical toxicities were measured at 3 and 6 months and reported using Common Terminology for Clinical Adverse Events (CTCAE) version 5 [24] (N.K., L. W., and B. C.). Treatment-related clinical toxicities included development of ascites, encephalopathy, and new-onset portal hypertensive changes including variceal formation. Biochemical toxicities included leukopenia, transaminitis, hyperbilirubinemia, and hypoalbuminemia.

Imaging response was evaluated by a board certified abdominal radiologist with 5 years of experience, blinded to the clinical trial, using mRECIST criteria at 6 months post Y90 [25] (A.D.)

### Statistical analysis

Receiver operating characteristic (ROC) analysis was used to determine what tumor dose and dosimetric variable predicted complete response (CR) or objective response (OR) as well as which non-tumoral liver dose (NTLD) predicted any clinical toxicity or severe, grade 3 or greater, treatment related toxicity. Comparisons between dosimetric measurements (segmental treatment vs non-segmental treatment) were performed using paired Student’s *t*-test and Pearson correlations. For treatment-related toxicities, chi square analysis was used. A *p*-value of 0.05 was used as the significance threshold. All statistics were performed using SAS software version 9.4 (SAS Institute, Cary, NC).

This cohort was previously described as a proof-of-concept study evaluating the accuracy of “scout” low-dose Y90 as a mapping agent compared to Tc99 MAA (PMID: 36,075,560) [10].

## Results

### Demographics

Thirty patients (*N* = 30) with 33 tumors were recruited and treated from December 2019 to January 2021. The cohort had a mean age of 66.4 years (standard deviation 6.6 years) and was comprised of 90% men (28 participants out of 30) (Table 1). Etiology of HCC was due to hepatitis C (17 participants, 55%), alcohol (7 participants 23%), non-alcoholic fatty liver disease (4 participants, 13%), and hepatitis B (4 participants, 13%). The mean model for end-stage liver disease (MELD) score was 10 (standard deviation 3.1). Most participants were Child–Pugh A (*n* = 24, 77%), albumin bilirubin (ALBI) score 1 (*n* = 17, 55%), ECOG 0 (*n* = 25, 81%), and BCLC A (*n* = 16, 53%). Mean tumor volume for the entire cohort was 44.9 cc (segmental mean volume 33.7 cc, SD 30.3 cc vs. non-segmental mean volume 58.4 cc, SD 92.7 cc, *p* > 0.05). Additionally, the mean volume of the segment/lobe of the liver perfused/treated with Y90 was 351.6 cc (segmental mean volume 226.5 cc, SD 114.2 cc vs. non-segmental mean volume 451.6 cc, SD 263.1, *p* = 0.025 (Table 1).

**Table 1:** Demographics of the entire cohort

	Frequency*	%	SD	Median
Mean age (years)	66.4		6.6	68
Male gender	28	93%		
Etiology of HCC				
Hepatitis B	4	13%		
Hepatitis C	17	57%		
EtOH	7	23%		
NAFLD	4	13%		
MELD	10		3	10
Child–Pugh				
A	24	80%		
B	6	20%		
ALBI score	− 2.62			
ALBI grade 1	17	57%		
ALBI grade 2	13	43%		
ECOG				
0	25	83%		
1	5	17%		
2	0	0%		
3	0	0%		
4	0	0%		
BCLC				
A	16	53%		
B	9	30%		
C	5	17%		
Tumor volume (cc)				
Entire cohort	44.9		52.1	
Segmental	33.7		30.3	
Non-segmental	58.4		92.7	
Liver volume (cc)				
Entire cohort	1737.1		397.2	
Segmental	1844.5		411.7	
Non-segmental	1614.4		452.9	
Perfused/treated non-tumoral liver volume (cc)				
Entire cohort	351.6		215.1	
Segmental	226.5		114.2	
Non-segmental	451.6		263.1	

^*^Frequency unless otherwise stated by use of parenthesis

### Mean tumor dose (TD) and non-tumoral liver dose (NTLD)

Of the 33 tumors, 19 were treated by radiation segmentectomy and 14 were treated in a non-segmental fashion. Specifically, 18 patients had segmentectomies and 12 patients had non-segmental treatments. Mean tumor dose for the entire cohort was 494 Gy (SD 344 Gy). Patients treated with segmental Y90 radioembolization had a higher mean tumor dose of 634 Gy compared to those treated with non-segmental Y90 who had a mean tumor dose of 304 Gy (*p* = 0.004) (Table 2). Mean value of dosimetric parameters for tumors calculated by 3D voxel-based dosimetry was significantly greater for those treated with segmental Y90 than those treated with non-segmental Y90 (TD-V30 segmental 761 Gy vs non-segmental 368 Gy, *p* = 0.004, TD-V70- segmental 522 Gy vs 248 Gy, *p* = 0.005, and minimum TD- segmental 236 Gy vs non-segmental 103 Gy *p* = 0.005).

**Table 2:** Mean TD and NTLD for the entire cohort, those treated with segmental Y90, and those treated with non-segmental Y90 (Bolded *p*-values denote statistical significance)

	Entire cohort *N* = 30	SD	Segmental *N* = 18	SD	Non-segmental *N* = 12	SD	*p*-value (segmental vs non-segmental)
TD							
TD-V30	594.1	405.1	760.5	418.4	368.3	257.2	**0.004**
Mean TD	493.7	343.7	633.9	354.9	303.5	220.6	**0.004**
TD-V70	405.7	288.1	522.3	297.9	247.6	186.1	**0.005**
Minimum TD	179.4	141.4	236.0	150.2	102.6	83.3	**0.005**
NTLD							
NTLD-V30	224.0	109.9	256.5	113.9	176.3	87.4	**0.048**
Mean NTLD	103.0	66.1	112.3	74.5	88.9	50.8	0.351
NTLD-V70	77.2	51.0	80.9	53.4	71.7	48.9	0.637
Minimum NTLD	24.1	33.8	27.2	32.0	19.5	37.2	0.555

Mean non-tumoral liver dose (NTLD), NTLD-V70, and minimum NTLD were not significantly different between the segmental and non-segmental groups (*P* > 0.05). However, NTLD-V30 was significantly higher in the segmental group (segmental 256 Gy vs 176 Gy, *p* = 0.048) (Table 2).

### Patient outcomes at 3 And 6 months

One patient died of disease progression within 30 days post Y90 and was excluded from the treatment response and toxicity analysis. At 3 months post Y90, objective response was achieved for 26 of the 32 treated tumors (81%). In the segmental group, all 18 (100%) had an objective response, while the non-segmental group only had 8 tumors (57%) for which objective response was achieved (*p* = 0.002). Complete response was observed for 23 (73%) tumors. More tumors in the segmental group (17 (94%) achieved complete response than the non-segmental group (6 (43%); *p* = 0.001)) (Table 3). There was no difference in objective and complete response rates at 6 months post Y90. Specifically, the same degree of partial and complete response was noted in the same patients and tumors at 6 months.

**Table 3:** Objective response, complete response, and clinical toxicities over the study period (Bolded p-values denote statistical significance)

	Entire cohort	%	Segmental	%	Non-segmental	%	*p* value (segmental vs non-segmental)
Tumor response (*N* = 32 tumors;18 segmental, 14 non-segmental)							
Objective response	26	81%	18	100%	8	57%	**0.002**
Complete response	23	72%	17	94%	6	43%	**0.001**
Adverse events (*N* = 29 patients; 17 segmental, 12 non-segmental)							
3 months: all grade AEs	23	79%	11	65%	12	100%	0.648
3 months: grade 3 AE	3	10%	0	0%	3	25%	0.071
6 months: all grade AEs	18	62%	8	47%	10	83%	0.455
6 months: grade 3 AE	4	14%	1	6%	3	25%	0.224

Treatment-related adverse events (AE) were prevalent, with 23 patients (79%) in the entire cohort experiencing any AE at 3 months and 18 (62%) experiencing any AE at 6 months. However, the majority of AEs were mild (grade 1 or 2): 20 patients (69%%) at 3 months and 14 patients (48%) at 6 months. Grade 3 toxicities were experienced by 3 patients (10%) at 3 months in the entire cohort and 4 participants (14%) of the entire cohort at 6 months. Any grade or severe clinical toxicities were not significantly different between the segmental and non-segmental groups (Table 3). No grade 4 or 5 toxicities were experienced.

The most common treatment-related mild clinical adverse events were fatigue (14% at 6 months and 7% at 6 months), nausea, vomiting, anorexia (14% at 3 months and 7% at 6 months), and abdominal pain (10% at 3 months and 6 months). There were no severe clinical adverse events at 3 months. At 6 months, 2 patients (7%) experienced grade 3 ascites (Table 4).

**Table 4:** Treatment-related clinical and biochemical adverse events at 3 and 6 months after Y90 radioembolization

	3 Months				6 Months			
	Grades 1–2	%	Grade 3	%	Grades 1–2	%	Grade 3	%
Clinical AEs	9	31%	0	0%	7	24%	2	7%
Fatigue	4	14%	0	0%	2	7%	0	0%
Nausea, vomiting, or anorexia	4	14%	0	0%	2	7%	0	0%
Abdominal discomfort	3	10%	0	0%	3	10%	0	0%
Ascites	1	3%	0	0%	1	3%	2	7%
Encephalopathy	1	3%	0	0%	1	3%	0	0%
Biochemical AEs	22	76%	3	10%	16	55%	2	7%
Decreased white blood cells	2	7%	2	7%	1	3%	0	0%
Decreased platelet count	12	41%	1	3%	8	28%	1	3%
Anemia	8	28%	0	0%	6	21%	0	0%
Increased AST or ALT	6	21%	0	0%	11	38%	0	0%
Increased ALP	2	7%	0	0%	1	3%	0	0%
Increased total blood bilirubin	5	17%	0	0%	4	14%	1	3%
Decreased albumin	0	0%	2	7%	2	7%	0	0%
Increased creatinine	2	7%	0	0%	2	7%	0	0%
Increased international normalized ratio	10	34%	0	0%	5	17%	0	0%
Hyponatremia	1	3%	0	0%	1	3%	0	0%
Any AEs	23	79%	3	10%	18	62%	4	14%

The most common treatment-related, biochemical, and mild AE at 3 months were decreased platelet count (41%), increased international normalized ratio (34%), and anemia (28%). The grade 3 severe biochemical AE at 3 months were decreased white blood cells (7%), decreased platelet count (3%), and decreased albumin (7%) (Table 4). At 6 months, the most common biochemical mild AEs were increased alanine or aspartate transaminase (38%), decreased platelet count (28%), and anemia (21%). The two severe biochemical AEs at 6 months were decreased platelet count (3%) and increased total blood bilirubin (3%) (Table 4).

### Tumor dose response thresholds

**Fig. 1:**
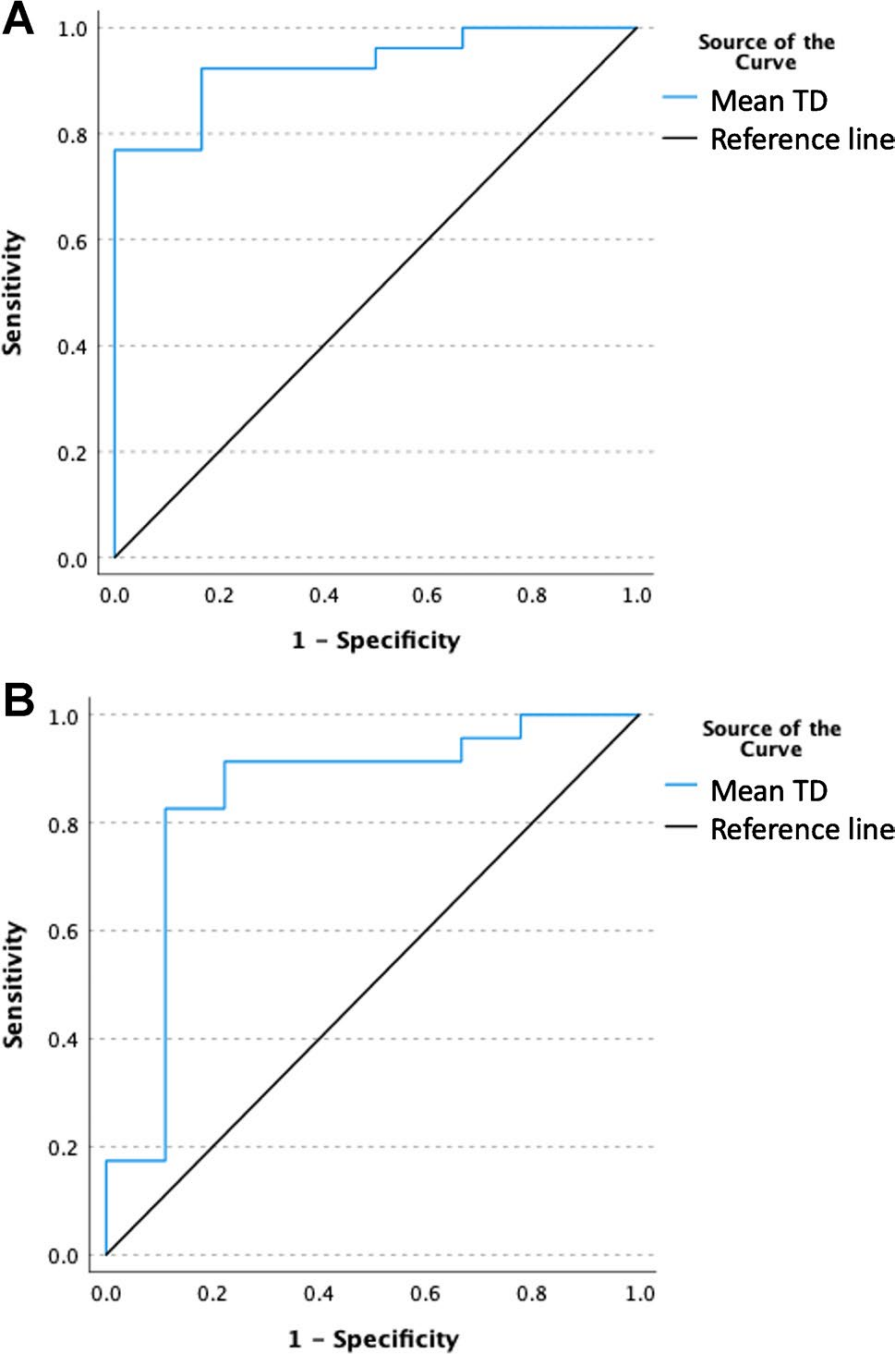
Tumor dose predicts objective response (**A**) and complete response (**B**) to Y90 radioembolization therapy

Mean tumor dose that predicted objective tumor response at 3 and 6 months with 92% sensitivity and 83% specificity was 253 Gy (AUC 0.929*, p* =  < 0.001) (Table 5, Fig. 1A). Mean tumor dose that predicted complete tumor response with 83% sensitivity and 89% specificity was 337 Gy (AUC 0.845, *p* =  < 0.001) (Table 5, Fig. 1B). All analyzed dose volume dosimetric factors, including mean TD-V30, mean TD-V70, and minimum TD, were found to be significant predictors of objective and complete responses, with areas under the curve (AUC) > 0.5 and *p* < 0.001 (Table 5).

**Table 5:** Tumor dose (TD) thresholds to predict tumor response to Y90 therapy and non-tumoral liver dose (NTLD) thresholds to predict severe clinical toxicity at 3 and 6 months after Y90 (Bolded *p*-values denote statistical significance)

		AUC	*P*-value	Threshold (Gy)	Sensitivity	Specificity
Tumor dose objective response ROC analysis						
TD-V30		0.929	** < 0.001**	306	92%	83%
Mean TD		0.929	** < 0.001**	253	92%	83%
TD-V70		0.917	** < 0.001**	213	85%	83%
Minimum TD		0.833	** < 0.001**	108	73%	67%
Tumor dose complete response ROC analysis						
TD-V30		0.836	**0.001**	359	91%	89%
Mean TD		0.845	** < 0.001**	337	83%	89%
TD-V70		0.831	** < 0.001**	258	83%	89%
Minimum TD		0.812	** < 0.001**	131	74%	89%
3 months: grade 3 toxicity						
NTLD-V30	Non-segmental	0.875	** < 0.001**	144	100%	75%
	Segmental	0.333	0.252			
Mean NTLD	Non-segmental	1.000	** < 0.001**	81	100%	100%
	Segmental	0.381	0.189			
NTLD-V70	Non-segmental	0.906	** < 0.001**	59	75%	100%
	Segmental	0.405	0.593			
Minimum NTLD	Non-segmental	0.875	** < 0.001**	20	75%	87%
	Segmental	0.571	0.598			
6 months: grade 3 toxicity						
NTLD-V30	Non-segmental	0.815	**0.016**	143	67%	67%
	Segmental	0.294	0.302			
Mean NTLD	Non-segmental	1.000	** < 0.001**	87	100%	100%
	Segmental	0.294	0.062			
NTLD-V70	Non-segmental	1.000	** < 0.001**	59	100%	100%
	Segmental	0.471	0.808			
Minimum NTLD	Non-segmental	0.926	** < 0.001**	20	100%	89%
	Segmental	0.765	0.241

### Non-tumoral liver dose toxicity thresholds

**Fig. 2:**
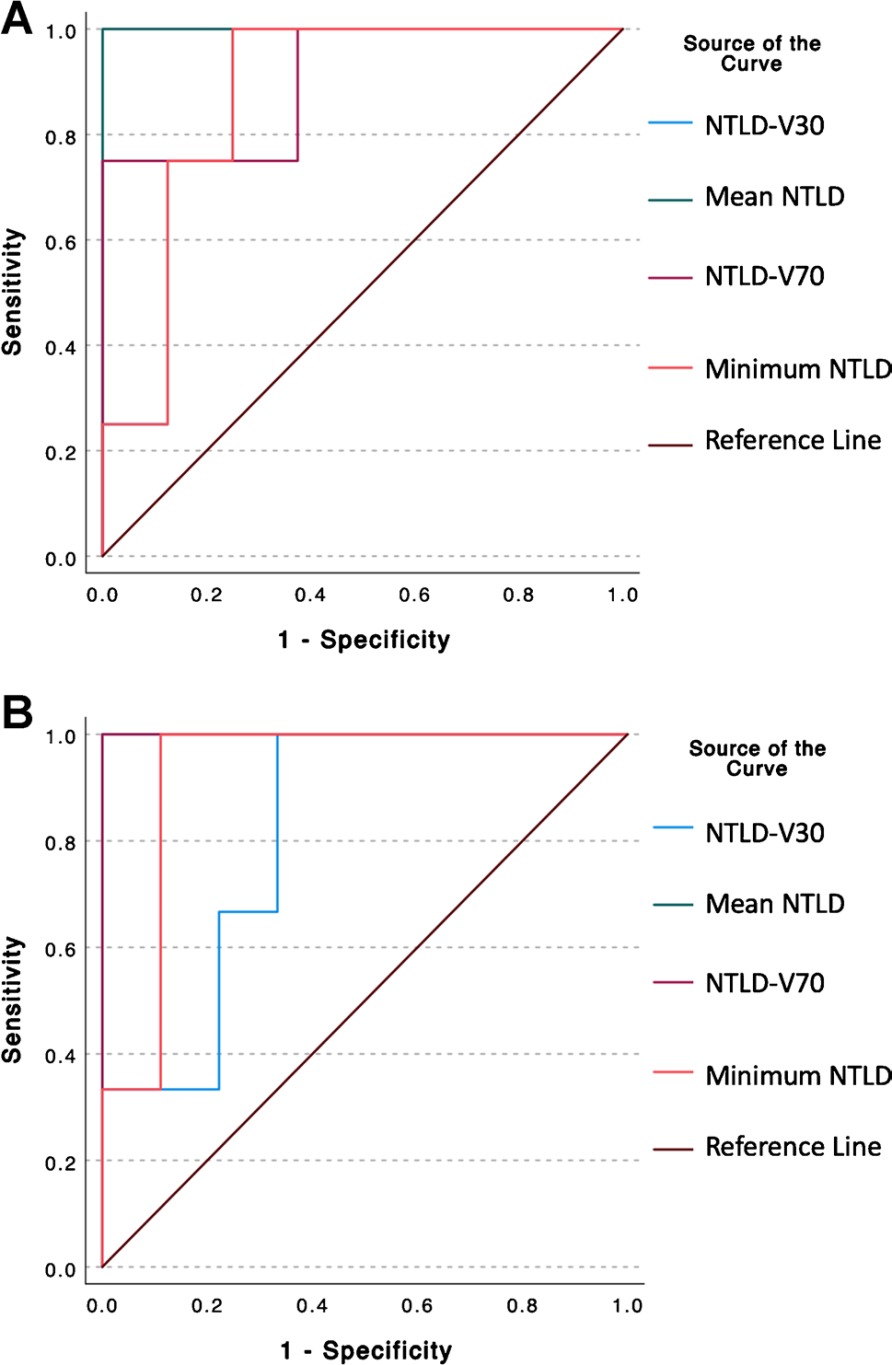
Non-tumoral liver dose (NTLD) predicts severe clinical toxicities at 3 months (**A**) and 6 months (**B**) after non-segmental Y90 radioembolization therapy

All analyzed dosimetric NTLD predicted serious grade 3 adverse events at 3 and 6 months for the non-segmental cohort (Table 5). Specifically, at 3 months, mean NTLD of 81 Gy or greater predicted a grade 3 adverse event with 100% sensitivity and 100% specificity (AUC = 1.000, *p* < 0.001) (Table 5, Fig. 2A). At 6 months, NTLD of 87 Gy or greater predicted grade 3 adverse events with 100% sensitivity and 100% specificity (AUC = 1.000, *p* < 0.0001) (Table 5, Fig. 2B). Additionally, minimum NTLD of 20 Gy also predicted grade 3 adverse events in the non-segmental cohort at 3 and 6 months. No significant NTLD thresholds predicted grade 3 adverse events for the segmental cohort (Table 5).

## Discussion

To date, there has been a paucity of resin-based Y90 dosimetry studies evaluating tumor dose response and toxicity thresholds in patients with HCC. This study attempted to evaluate treatment response and toxicity in patients with treatment naïve HCC treated with resin microspheres in a prospective fashion. It showed that complete and objective responses can be predicted by Y90 tumor doses greater than 337 and 253 Gy, respectively. Additionally, serious adverse events can be predicted from non-segmental Y90 radioembolization with non-tumoral liver doses exceeding 80 Gy.

These dosimetric findings are important to improve patient response to therapy and ultimately patient survival [2]. The DOSISPHERE-01 trial found that personalized dosimetry with tumor doses > 205 Gy was associated with greater response rate without significantly more adverse events in patients with HCC treated with glass-based Y90 [2]. Another retrospective study found that 400 Gy tumor dose with glass-based Y90 was associated with complete pathologic necrosis in HCC [5]. The RASER study examined radiation segmentectomy in early stage, unresectable HCC with a median tumor dose of 1004 Gy associated with a 90% response rate when treated with glass-based Y90 [3]. The post-hoc dosimetry analysis of the SARAH trial demonstrated that in patients with HCC treated with resin-based Y90, the probability of disease control was 72%, 81%, and 90% for mean tumor radiation-absorbed doses of 100, 120, and 150 Gy, respectively [26]. Additionally, in the SARAH trial, overall survival was significantly greater in patients receiving 100 Gy or higher compared to others. To the authors’ knowledge, the current study is the first prospective investigation of tumor dose response thresholds in HCC patients treated with resin-based Y90 radioembolization. The study found a threshold of 337 Gy to predict a complete response after single treatment. We believe that the significant discrepancy found between the tumor dose response thresholds found in our study versus the post-hoc analysis of the SARAH trial has to do with several factors: (1) in the current study, the tumor dose to achieve objective and complete imaging responses was evaluated, whereas in SARAH data, thresholds to achieve disease control were reported; (2) the activity calibration for the SARAH trial was the day of calibration with significantly less specific activity per resin microsphere compared to the 2- and 3-day pre-calibrated resin microspheres used in our study. This in turn resulted in higher number of administered particles per prescribed activity in the SARAH trial compared to the current study. We hypothesize that these differences led to the significantly higher tumor dose response thresholds observed in the current study compared to that of the SARAH trial.

In addition to tumor dose, this study also evaluated a dosimetric threshold of non-tumoral liver dose to predict adverse events after Y90 radioembolization. Multiple studies have been unable to ascertain a dose due to rare grade 3 or higher adverse events. This includes the recently published TARGET study, which was a retrospective investigation of 209 patients with HCC who were treated with glass-based Y90, that was unable to find a relationship between NTLD and hyperbilirubinemia [4]. Other studies have also failed to find NTLD thresholds in metastatic disease to predict serious adverse events [27]. A retrospective review by Chiesa et al. found that in patients with HCC treated with glass-based Y90 in a lobar fashion, a NTLD of 75 Gy predicted 15% risk of clinical toxicity, which included change in Child–Pugh class [28]. NTLD threshold is an important dosimetric factor to study because serious adverse events after Y90 can be life-threatening. In this study, NTLD of 81 Gy or higher predicted grade 3 at 3-month post Y90 and NTLD of 87 Gy or higher predicted grade 3 AEs at 6-month for patients treated in the non-segmental fashion. No dose threshold was found for the segmental fashion. This reported threshold is significantly higher than the mean 40 Gy threshold recommended by a recent international consensus panel for personalized treatment planning with Y90 resin microsphere [29]. However, the aforementioned recommendation was explicitly based on external beam radiotherapy data using biologic effective dose. Additionally, the 40 Gy threshold recommendation is based dose to entire non-tumoral liver volume as apposed the treated non-tumoral liver volume reported in this study. Interestingly, in the current study, a minimum NTLD of 20 Gy also predicted grade 3 adverse events in the non-segmental cohort at 3 and 6 months [30]. While the thresholds reported in the current study can be used as benchmark for more personalized treatment planning for patients with HCC treated with resin-based Y90, they need to be confirmed using a larger pool of data.

The recently published RASER study reported 8 (28%) grade 3 serious treatment-related adverse events in their 29 patients with HCC treated with glass-based Y90 radiation segmentectomy [3]. Other retrospective, studies also reported frequent adverse events after Y90 including 48% with fatigue and 38% with abdominal pain, and laboratory toxicities which were generally mild (grades 1–2) and transient [7, 8]. These reported adverse events are concordant with the rates of AEs in this study with mild adverse events occurring in 68% of the patients at 3 months and 62% of the patients at 6 months. The serious AEs observed in this study were reported in 10% of the patients at 3 months and 14% of the patients at 6 months. Additionally, the proportion of grade 3 AEs was higher in the non-segmental therapy cohort both at 3 and 6 months, though not statistically significant.

Limitations of this study include its small number of 30 patients, which was further divided into segmental and non-segmental therapies. It is possible that with more power, a NTLD threshold may have been found for the segmental cohort as well, albeit less likely due to limited area of the liver being treated in segmentectomies. Additionally, this study was conducted at a single center, which may not reflect real-world practice technique variations. The study aimed to deliver less than 70 Gy to the NTL; however, there were cases where higher doses were delivered. This discrepancy is likely due to inherent inaccuracies of prediction of Y90 biodistribution post therapy versus Tc99 MAA, which was used for the prospective treatment [10, 12]. Our segmental cohort had a 3-day pre-calibration Y90 activity vs a 2-day precalibration for the non-segmental group. This affected the approximate number of microspheres delivered with slightly less radioactivity in each microsphere. Because the exact same activities were not delivered per sphere, the comparison between the segmental and non-segmental approaches may be confounded due to a greater number of microspheres given in the non-segmental approach. This trial was single armed and not randomized into multiple arms of varying NTLD and TD. Variation in NTLD arose from treatment planning and patient biological differences and thus was not an independently controlled variable because patient tumor dosages were prescribed per the best available evidence at the time of the inception of this study. The clinical follow-up of 6 months limits the authors’ ability to assess longer-term clinical toxicities.

This work suggests that in patients with HCC treated with 2- or 3-day pre-calibrated resin-based Y90 radioembolization, mean target tumor dose should be greater than 337 Gy to achieve complete imaging response. However, and specific to non-segmental treatment, mean non-tumoral liver dose should be planned to be less than 80 Gy in order to minimize serious treatment-related toxicity. Future, larger, randomized, blinded trials will be needed to validate tumor dose and non-tumor liver dosimetric thresholds to maximize tumor response while minimizing clinical adverse events in patients with surgically unresectable hepatocellular carcinoma.
